# Mesenchymal Stem Cells in Nerve Tissue Engineering: Bridging Nerve Gap Injuries in Large Animals

**DOI:** 10.3390/ijms24097800

**Published:** 2023-04-25

**Authors:** Mirko Lischer, Pietro G. di Summa, Ilias G. Petrou, Dirk J. Schaefer, Raphael Guzman, Daniel F. Kalbermatten, Srinivas Madduri

**Affiliations:** 1 Center for Bioengineering and Regenerative Medicine, Department of Biomedical Engineering, University of Basel, 4123 Allschwil, Switzerland; 2 Department of Plastic, Reconstructive and Hand Surgery, University Hospital of Lausanne and University of Lausanne, 1015 Lausanne, Switzerland; 3 Plastic, Reconstructive and Aesthetic Surgery, Department of Surgery, University Hospitals and University of Geneva, 1205 Geneva, Switzerland; 4 Department of Plastic, Reconstructive, Aesthetic and Hand Surgery, University Hospital Basel, University of Basel, 4031 Basel, Switzerland; 5 Department of Neurosurgery, University Hospital Basel, 4031 Basel, Switzerland; 6 Bioengineering and Neuroregeneration, Department of Surgery, Geneva University Hospitals and University of Geneva, 1205 Geneva, Switzerland

**Keywords:** cell therapy, large animal models, nerve injury, nerve regeneration, nerve guidance conduit, Schwann cells, stem cells, growth factors

## Abstract

Cell-therapy-based nerve repair strategies hold great promise. In the field, there is an extensive amount of evidence for better regenerative outcomes when using tissue-engineered nerve grafts for bridging severe gap injuries. Although a massive number of studies have been performed using rodents, only a limited number involving nerve injury models of large animals were reported. Nerve injury models mirroring the human nerve size and injury complexity are crucial to direct the further clinical development of advanced therapeutic interventions. Thus, there is a great need for the advancement of research using large animals, which will closely reflect human nerve repair outcomes. Within this context, this review highlights various stem cell-based nerve repair strategies involving large animal models such as pigs, rabbits, dogs, and monkeys, with an emphasis on the limitations and strengths of therapeutic strategy and outcome measurements. Finally, future directions in the field of nerve repair are discussed. Thus, the present review provides valuable knowledge, as well as the current state of information and insights into nerve repair strategies using cell therapies in large animals.

## 1. Introduction

Peripheral nerve defects can have a devastating impact on patients’ lives due to loss of function and, consequently, lifelong disability [1]. The current gold standard treatment for the surgical repair of nerve gap injuries is autologous nerve grafting [1,2]. This method leads to acceptable results, but it is associated with several disadvantages. Among these, the sacrifice of a healthy nerve and the corresponding morbidity of the donor site are the most severe. By contrast, nerve allografts result in immune rejection, thus requiring systemic immunosuppressive drug treatment [3]. In the search for better solutions, synthetic nerve conduits (NGCs) or tissue-engineered nerve grafts (TENGs) have emerged [2]. TENGs are usually composed of a biodegradable or biocompatible scaffold, which are widely enriched with support cells and bioactive substances or growth factors [4] designed for creating an optimal microenvironment for faster and more effective nerve regeneration [2,5,6].

Hu et al. describe the role of mesenchymal stem cells (MSCs) as a potential source of therapeutic growth factors for TENGs [7]. The ideal source of therapeutic cells should enable easy access and rapid proliferation and differentiation under controlled conditions. Within this context, MSC can differentiate into Schwann cell-like cells (SCLC) under specific stimuli and promote peripheral nerve regeneration [8,9,10,11]. MSCs with a multi-lineage capacity can be isolated from multiple sources, such as bone marrow, adipose tissue, dental pulp, umbilical cord blood, skin tissue, and amniotic fluid [12,13,14,15]. They can rapidly proliferate under controlled conditions [16] with a multi-lineage capacity, i.e., adipocytes, osteoblasts, and chondrocytes [13,17,18] over a prolonged culture time.

MSCs possess a neurotrophic and neuritogenic capacity to support nerve regeneration. The neurotrophic potential of MSCs is attributed to its secretome containing a wide range of biochemical and molecular factors [19]. MSC exosomes releasing miRNA21, miRNA222, and miRNAlet7a play an important role in nerve plasticity and regeneration. Furthermore, the secretome of MSCs is rich with various growth factors such as nerve growth factor (NGF), brain-derived neurotrophic factor (BDNF), neurotrophin-3 (NT-3), glial cell-derived neurotrophic factor (GDNF), insulin-like growth factor 1 (IGF-1), vascular endothelial growth factor (VEGF), epidermal growth factor (EGF), basic fibroblast growth factor (bFGF), transforming growth factor beta (TGF-ß), and platelet-derived growth factors (PDGF) [20,21,22,23,24], which are directly linked to axonal growth and elongation. Moreover, transplanted MSCs may possess paracrine activity on the endogenous peripheral glial cells boosting the process of nerve regeneration [25]. In allogeneic transplantation, the use of MSCs greatly benefits from their hypo-immunogenicity or immune evasion by virtue of the reduced expression of human leukocyte antigen (HLA)-DR class II histocompatibility antigen [26,27]. Thus MSCs, among various types of support cells, represent a potential therapeutic source for TENGs for the treatment of nerve injuries. Similar to neural stem cells (NSCs), MSCs are able to differentiate into neuronal progenitor cells [8,28], which have the capacity to enhance nerve regeneration [7,29,30]. Although SCs can also be used to achieve the same outcome [31], obtaining autologous SCs would imply the use of a nerve biopsy and, therefore, the sacrifice of a healthy nerve. Moreover, their culture is difficult and time-consuming, thus limiting the use of SCs in clinical settings on a ready-to-use basis [32].

The development of TENGs is an emerging field with a huge potential for treating severed nerve injuries and eventually providing viable options for autologous nerve grafting. Within this context, several approaches involving various artificial or natural NGCs in combination with different sources of support cells, particularly MSCs, have been reported in the literature. The outcome measurements were evaluated using different types of animal models [33,34,35,36,37,38,39]. As opposed to small animal models, large animal experimental models provide a critical and comparable microenvironment to that of human nerve injuries and the associated regeneration process. The purpose of this systematic review was to gain a comprehensive overview of various approaches involving NGCs and stem cells that have reached a more advanced stage of therapeutic evaluation by using large animal models such as rabbits, dogs, pigs, sheep, and monkeys.

## 2. Methods

### 2.1. Eligibility Criteria

A systematic review was conducted for articles published before December 2022 according to the Preferred Reporting Items for Systematic Reviews and Meta-Analyses (PRISMA) guidelines. The review included original peer-reviewed studies based on the following inclusion criteria: (1) publication in an English-language journal; and (2) in vivo animal studies evaluating peripheral nerve repair using stem cells in large animals, such as rabbits, dogs, swine, sheep, and non-human primates. One clinical trial was also included. Exclusion criteria were studies involving lower-order animals such as rodents. Another study was excluded because the primary endpoint was not relevant for axonal regeneration and concerned the in vivo tracking of cells using magnetic resonance imaging [40]. Letters, editorials, review articles, patents, conference and meeting abstracts were excluded.

### 2.2. Search Strategy and Study Selection

We conducted a literature search using PubMed, EMBASE, and Medline databases. The title and abstract field were selected to search for key terms pertaining to nerve repair, long-gap nerve injuries, cell therapy, and large animal models of nerve injury. Studies identified by the search outcomes were combined, and duplicates were excluded. Screening of the title and abstracts was performed before the extraction of full-text articles. The search yielded 224 studies. Of these, 194 were removed. The remaining studies underwent final full-text review and were confirmed as meeting the inclusion criteria. The underlying reason behind the low number of studies (about 30) matching the search criteria is the limited number of large animal studies involving gap injuries and cell-based therapeutic strategies. Furthermore, the increased number of complex ethical and regulatory requirements to conduct large animal studies using cell-based nerve guidance conduits may also explain the scarce number of available studies. Selected articles were categorized based on animal model, type of nerve guidance scaffold, and source of stem cells applied to regenerate gap nerve injuries in large animal models.

## 3. Results

**Figure 1:**
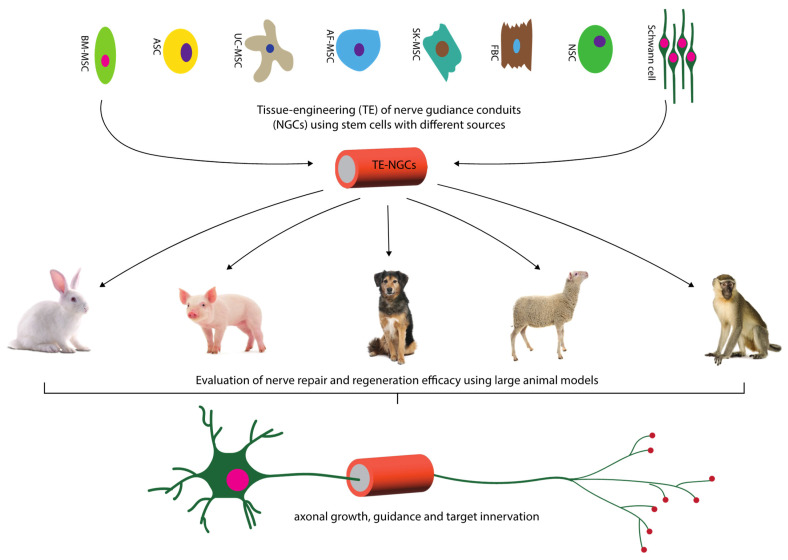
Tissue engineering of nerve guidance conduits using different MSCs and other cells. TE-NGCs were tested for bridging the nerve gap injuries in various large animal models covering rabbit, pig, dog, sheep and monkey. Therapeutic efficacy of these cell-based therapies was assessed measuring histomorphometry, electro physiological properties and behavioral recovery. Key: BM-ASC, Bone marrow-derived mesenchymal stem cells; ASC, Adipose-derived stem cells; UC-MSC, Umbilical cord-derived MSCs; AF-MSC, Amniotic fluid-derived MSCs; SK-MSCs, Skin-derived MSCs; FBC, Fibroblast cells; NSC, Neural stem cells.

Selected publications reporting the use of cell-based engineered nerve guidance grafts were reviewed and categorized based on the type of materials used and the source of stem cells (Figure 1).

### 3.1. Scaffold Materials Used for Nerve Guidance Grafts

A large variety of scaffolds have been used in peripheral nerve regeneration research and can be grouped approximately into three different categories: biocompatible (but not biodegradable) artificial materials; biodegradable artificial materials; and materials of biological origin. The main function of these scaffolds is to guide the regenerating nerve fibers, protect the regenerating nerve from outer influences, and keep the neurotrophic factors in place [41].

Biocompatible artificial scaffolds, such as natural polymers [39], silicone tubes [42,43], and ePGFE (GoreTex^®^) tubes [41], are easy to produce and sterilize. However, depending on the location and size of the repaired nerve gap, they may be uncomfortable for the patient and need to be surgically extracted [43]. This disadvantage of a foreign body sensation does not occur if the implant is biodegradable and thus vanishes over time. Examples of such scaffolds are chitosan conduits, which are used either alone [44] or in combination with poly(lactic-co-glycolic acid) (PLGA) fibers [4,43,45,46] or collagen [47]. Another artificial biodegradable conduit used is made of poly L-lactic acid and ε-caprolactone copolymer (PLAC) and is also filled with collagen [48,49,50].

Biological tissues can be used as scaffolds for peripheral nerve repair, with two main kinds of tissues reported, i.e., autologous and heterologous tissue grafts. The advantage of using autologous tissues is obviously the low immunogenicity and no risk of rejection, easy accessibility, and only minimal donor site morbidity. Autologous veins have been used as conduits, again filled with collagen [29], fibrin [51], or saline [52]. Experiments with acellular allogenic nerve grafts have also been performed in an attempt to develop an alternative solution [36,37,38,39]. Apart from the fact that the surgeon does not have to sacrifice a patient’s healthy nerve, the advantage of the latter technique is the reduced immunogenicity due to decellularization with a preserved basal lamina structure, thus giving the regenerating nerve the most natural possible scaffold [7,36,37,38,39,53,54]. Viable options for autologous nerve grafting include artificial nerve guidance grafts consisting of natural or synthetic biopolymers. The most commonly-used fabrication methods include spinning mandrel technology, gel spinning, film casting and rolling, molding and freeze-drying, electrospinning, and 3D bioprinting. Further advanced methods include the incorporation of guidance fibers, nano-micro patterns or grooves, growth factors, cytokines, DNA, miRNAs, and cells [55].

### 3.2. Stem Cells Used for Nerve Repair

Due to the immunomodulatory and pro-regenerative functions of stem cells, it is possible to transplant allogenic stem cells with a reduced risk for immunological rejection of the graft [47]. Since it is nearly impossible to obtain autologous neural stem cells (NSCs) without damaging the brain, NSCs may not be the desired source for clinical use. The two experiments reported with these cells were based on an allogenic source. Other xenogeneic sources of stem cells evaluated in research projects include human umbilical cord-derived stem cells (HUC-MSCs), human amniotic fluid stem cells (AF-MSC), human bone marrow-derived stem cells (BMSCs), human adipose-derived stem cells (ASCs) and MSC-derived SCLC (Table 1).

#### 3.2.1. Neural Stem Cells for Treating Peripheral Nerve Injuries of Large Animals

Guo and Dong adopted xenografted NSCs from guinea pigs, which they seeded in a chitosan/PLGA conduit and implanted in a 10 mm facial nerve defect in rabbits. As a control, they used the empty conduit and nerve autografts. They observed that NSCs significantly enhanced nerve regeneration compared to the empty conduit, with no significant difference between the chitosan-based TENG and the autograft [47]. Cheng et al. used NSCs in a 10-mm-long traction injury of the sciatic nerve in rabbits and attempted to assess regeneration with magnetic resonance imaging (MRI). They concluded that the gadopentetate-dimeglumine-labeling technique allows the tracking of NSCs in vivo. The MRI observation was confirmed by histology with improved recovery in injured nerves with NSCs, provided that histologic sections of the regenerated nerve did not show NSC differentiation to SCs, thus suggesting that the regenerative effect of NSCs might be paracrine through neurotrophic factors [56]. However, the paucity of publications with NSCs and the different kinds of injury reported in these two reports does not allow any complete conclusions. Nevertheless, both groups receiving NSCs showed a significant improvement over controls, and there is, therefore, the scope for NSC-based cell therapy for treating clinical nerve gap injuries, but further studies are needed to evaluate the functional and behavioral benefits.

**Table 1:** Repair and reconstruction of sub-critical and critical nerve injuries in large animals: strategies involving various scaffolding materials and cell sources.

Author	Animal Species	Nerve Graft Composition	Cell Source	Experimental Model	Follow-Up (Days)
Choi et al., 2005 [29]	Rabbit	Autologous vein/collagen	BMSCs	15 mm peroneal nerve defect	90
Hu et al., 2007 [7]	Rhesus macaque	Acellular allogenic nerve	BMSCs	40 mm ulnar nerve defect	180
Braga et al., 2008 [43]	Human	Silicone tube	BMSCs	20–50 mm nerves in forearm	360
Wang et al., 2008 [54]	Rhesus macaque	Acellular allogenic nerve	BMSCs	10 mm radial nerve defect	60
Guo et al., 2008 [47]	Rabbit	Chitosan/collagen	NSCs	10 mm facial nerve defect	120
Ding et al., 2010 [4]	Dog	Chitosan/PLGA	BMSCs	50 mm sciatic nerve defect	180
Wakao et al., 2010 [48]	Crab-eating macaque	PLAC/collagen	BMSCs-SCLCs	20 mm median nerve defect	360
Wang et al., 2010 [53]	Rhesus macaque	Acellular allogenic nerve	BMSCs	25 mm radial nerve defect	150
Shen et al., 2010 [57]	Rabbit	-	BMSCs	10 mm sciatic nerve defect	15
Cheng et al., 2011 [56]	Rabbit	(None)	NSCs	10 mm sciatic nerve defect	75
Wang X. et al., 2011 [52]	Rabbit	Autologous vein	SCLCs- BMSCs	10 mm facial nerve defect	150
Park et al., 2012 [58]	Pig	Collagen/fibrin	SK-MSCs	10 mm femoral nerve defect	30
Xue et al., 2012 [45]	Dog	Chitosan/PLGA	BMSCs	60 mm sciatic nerve defect	360
Duan et al., 2012 [40]	Rabbits	-	MSCs	10 mm sciatic nerve defect	70
Hara et al., 2012 [59]	Cynomolgus monkeys	Nerve lengthening	-	20 mm median nerve defect	112
Ghoreishian et al., 2013 [41]	Dog	ePTFE/alginate hydrogel	ASCs	7 mm facial nerve defect	120
Hu et al., 2013 [46]	Rhesus macaque	Chitosan/PLGA	BMSCs	50 mm median nerve defect	360
Casañas et al., 2014 [50]	Sheep	PLAC	BMSCs	10 mm tibial and radial nerve defect	180
Wang et al., 2014 [60]	Rhesus macaque	Acellular allografts	BMSCs	25 mm radial nerve defect	150
Lasso et al., 2015 [51]	Rabbit	Autologous vein/fibrin	ASCs	4 0 mm peroneal nerve defect	90
Trindade et al., 2015 [42]	Rabbit	Silicone tube	BMSCs	5 mm femoral nerve defect	75
Xiao et al., 2015 [44]	Rabbit	Chitosan	UC-MSCs	Peroneal end-to-side anastomosis	120
Kaizawa et al., 2016 [49]	Dog	PLAC, artery	BMSCs	30 mm ulnar nerve defect	180
Jiang et al., 2016 [61]	Rhesus macaque	Acellular nerve allografts	Allogeneic Schwann cells	40 mm ulnar nerve defect	150
Su et al., 2018 [62]	Mini-pigs	PLA conduit	hAF-MSCs	15 mm sciatic nerve defect	600
Cui et al., 2018 [63]	Dogs	Longitudinally- oriented collagen conduit	hUC-MSCs	35 mm sciatic nerve defect	270
Sun et al., 2018 [64]	Rabbits	Xenografts and autografts	ASCs and PRP	10 mm facial nerve defect	56
Mitsuzawa et al., 2019 [65]	Beagle dogs	Scaffold-free 3D conduits	Dermal FBCs	5 mm ulnar nerve defect	70
Fadia et al., 2020 [66]	Rhesus macaque	Poly (caprolactone) conduit and median nerve autograft	Micro-particles releasing GDNF	50 mm median nerve defect	365
Daradka et al., 2021 [67]	Mongrel dogs	Autologous saphenous vein graft	Autologous PRP BMSCs	10 mm facial nerve defect	56
Kornfeld et al., 2021 [39]	Sheep	Silk fibroin NGCs	ECM	60 mm tibial nerve defect	180
Contreras et al., 2022 [37]	Sheep	Acellular nerve allograft	ECM	50 mm/70 mm peroneal nerve defect	270
Contreras et al., 2023 [36]	Sheep	Acellular nerve allograft	ECM	70 mm peroneal nerve defect	270
Holzer et al., 2023 [38]	Rhesus macaque	Acellular nerve xenograft	ECM	40 mm radial nerve defect	360

Key: MSC, mesenchymal stem cells; BMSC, bone marrow-derived mesenchymal stem cells; NSC, neural stem cells; PLGA, poly(lactic-co-glycolic acid; PLAC, poly L-lactic acid and ε-caprolactone copolymer; SCLC, Schwann cell-like cells; ASC, adipose-derived stem cells; GDNF, glial cell-derived neurotrophic factor; PRP, platelet-rich plasma; NGC, nerve guidance conduit; ECM, extracellular matrix.

#### 3.2.2. Bone Marrow-Derived Stem Cells Promote Nerve Regeneration in Large Animals

A large number of studies in larger animal nerve repair have mainly focused on BMSCs, although the experiments differed in terms of animals, nerve gap size, and the type of NGC materials.

Choi et al. were the first to use MSCs for peripheral nerve repair in larger animals. To bridge a 15 mm peroneal nerve gap in rabbits, they used an autologous vein filled with collagen gel and autologous BMSCs—some of which had already differentiated in vitro to SCLC. After 24 weeks of treatment, animals treated with BMSCs exhibited a high number of myelinated axons with an increased diameter [29]. Two years later, Hu et al. were the first to use rhesus monkeys in 2007. They worked with acellular allogenic nerves seeded with autologous BMSCs to bridge a 40 mm ulnar nerve defect. After 6 months, they found significantly better electrophysiological readings of BMSC-seeded grafts compared to empty grafts. Notably, no significant difference was found between the autograft, SC graft, and BMSC graft. Together with the absence of adverse events, they concluded that acellular allogenic nerve grafts with BMSCs might serve as an autograft replacement. In 2008 and 2010, Wang et al. performed similar experiments but with smaller radial nerve defects of 10 mm and 25 mm, respectively, with a shorter follow-up. In addition, they assessed nerve regeneration with immunofluorescence staining for S-100 protein, an SC-specific marker, and marked the BMSCs with BrdU in 2008 and conducted a behavioral analysis in 2010. The results were again promising and showed that BMSCs differentiate in vivo into SCLCs and, consequently, the resulting outcome measurement compared well to animals treated with SCs, with an almost complete functional recovery [53,54].

Braga-Silva et al. were the first to investigate the clinical use of autologous BMSCs. In a retrospective study of median and ulnar nerve injury, patients treated with a BMSC-filled silicone tube from 2002 to 2007 were compared with those who had undergone silicone tube grafting only from 1992 to 1996. Although findings showed a significantly better recovery with BMSC-filled tubes [43], this was a retrospective study with a large time interval between the two groups, and improved surgical techniques may have had an impact on these results.

**Figure 2:**
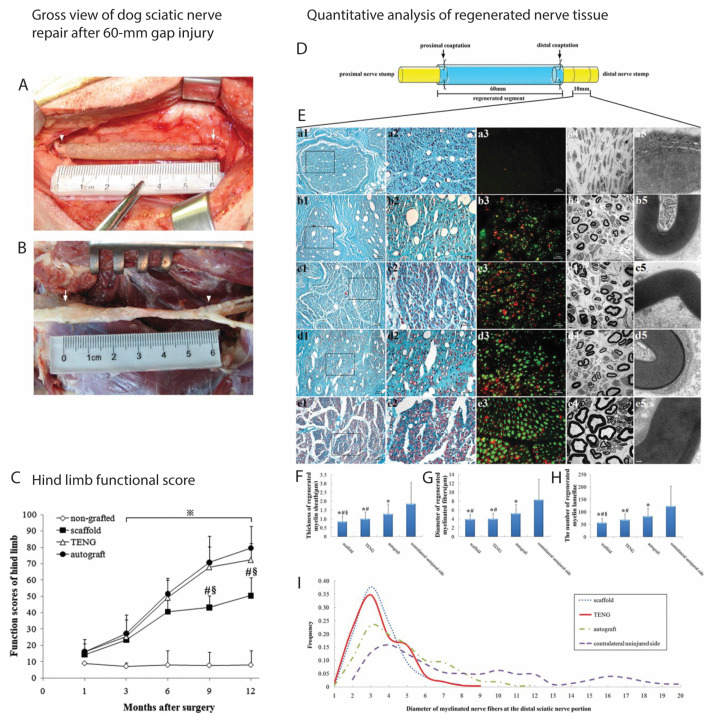
Dog sciatic nerve repair and regeneration using tissue engineered nerve graft consisting of chitosan/PLGA and autologous bone marrow mesenchymal stem cells. Gross view of dog sciatic nerve repair after 60 mm gap injury: View obtained immediately (**A**) and 12 months (**B**) after a tissue-engineered nerve graft was used to bridge a 60 mm gap in dog sciatic nerve. The proximal and the distal coaptations are indicated by an arrow and an arrowhead, respectively. Minimal scale: 1 mm; (**C**) Hind limb functional values represent means ± standard deviations. Two-way ANOVA, in which one factor was time points and the other was grouping, and post hoc Bonferroni *t* test were used to analyze the data. ^#^ *p* < 0.05 versus autograft group, ^§^ *p* < 0.05 versus tissue-engineered nerve graft (TENG) group, and ^※^ *p* < 0.05 versus nongrafted group; (**D**) showing the regenerated nerve segment in different grafted groups and (**E**) the distal portion from which nerve sections were harvested. Meyer trichrome staining (**a1**–**e1**,**a2**–**e2**), immunohistochemistry with anti-NF and anti-S-100 (**a3**–**e3**), transmission electron micrographs (**a4**–**e4**,**a5**–**e5**), obtained at 12 months postsurgery, of the sectioned regenerated nerve on the injured side in nongrafted (**a1**–**a5**), scaffold (**b1**–**b5**), tissue-engineered nerve graft (TENG; (**c1**–**c5**)), and autograft (**d1**–**d5**) groups and on the contralateral uninjured side (**e1**–**e4**), respectively. The higher magnifications of the boxed areas in (**a1**–**e1**) are shown in (**a2**–**e2**), respectively. Scale bar: 50 µm (**a1**–**e1**), 20 µm (**a2**–**e2**,**a3**–**e3**), 5 µm (**a4**–**e4**), 0.2 µm (**a5**–**e5**). Histograms showing the thickness of regenerated myelin sheath (**F**), the diameter of regenerated myelinated nerve fibers (**G**), the number of regenerated myelin lamellae (**H**) and the frequency distribution of myelinated nerve fiber diameters on the distal portion (**I**). All data are expressed as means ± standard deviations. One-way ANOVA plus post hoc Scheffé test for (**F**–**H**) and Kolmogorov–Smirnov test alone for (**I**) were used to analyze the data. * *p* < 0.05 versus the contralateral uninjured side, ^#^ *p* < 0.05 versus autograft group, and ^§^ *p*< 0.05 versus TENG group. These figures were adapted from published work by Xue et al., 2011 [45].

Further research by Ding et al. crossed the longest nerve gap injury so far (50 mm of dog sciatic nerve) using chitosan/PLGA NGC seeded with BMSCs and observed a regeneration close to the performance of autografts [4]. In a similar experiment involving a 60 mm nerve gap injury, a significant difference after one year was observed between the autograft and TENG NGCs. However, the TENG consisting of chitosan/PLGA NGCs and BMSCs was still significantly better than empty NGCs (Figure 2) [45]. Subsequently, these authors applied the resulting TENGs to rhesus monkeys. After one year, the 50 mm median nerve defects bridged with TENGs composed of BMSC-filled chitosan/PLGA NGCs showed a similar performance to that of autografts and were significantly better than empty NGCs. Furthermore, the blood test and histopathological analysis showed no anomalies of nerve regeneration, thus indicating the clinical potential of the BMSC-based TENGs [46].

**Figure 3:**
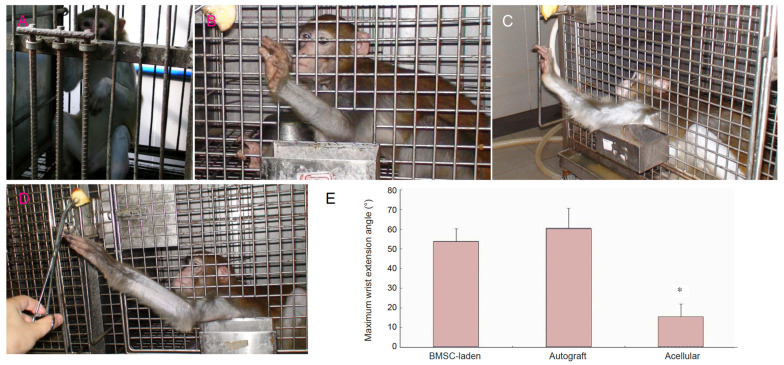
Repair and regeneration of rhesus monkey radial nerve 25 mm gap injury using autologous bone marrow mesenchymal stem cells. Behavioral assessment of the peripheral nerve 5 months after surgery. All animals displayed a lack of wrist extension after the radial nerve surgery (**A**). Five months after the surgery, the monkeys implanted with the autograft (**B**) and BMSC-laden allografts (**C**) showed a remarkable restoration of wrist-extension function. However, the animals that received acellular allografts exhibited reduced wrist-extension performance, with a smaller maximum wrist extension angle (**D**). (**E**) A smaller maximum wrist extension angle was seen in the acellular group compared with the BMSC-laden and autograft groups. * *p* < 0.05, vs. BMSC-laden and autograft groups (n = 6 forearms). Data are expressed as mean ± SD. One-way analysis of variance with the Student-Newman-Keuls multiple comparisons method was used for statistical testing. BMSC: Bone marrow stem cell. These figures were adapted from published work by Wang et al., 2014 [60].

In a different study involving a 25 mm long radial nerve defect in a rhesus monkey, an acellular allogeneic nerve graft functionalized with autologous BMSCs was evaluated. As a control, an acellular graft or autologous nerve graft was implanted. NGCs engineered with BMSCs resulted in a remarkable functional recovery at 5 months follow-up, as evidenced by axonal regrowth and myelinated fiber density in the distal stump of the radial nerve. These observations were coupled with an enhanced function in wrist extension, nerve conduction, and amplitude (Figure 3) [60].

In contrast to the previous authors who transplanted BMSCs without processing, Wakao et al. used BMSCs that differentiated in vitro to an SC-like phenotype. In a one-year trial with cynomolgus monkeys, they repaired a 20 mm median nerve gap with PLAC conduits seeded with BMSC-derived SCLCs. They observed a significantly better regeneration of axons and functionality in seeded grafts than in empty grafts, thus indicating that BMSC-SCLCs, similar to undifferentiated BMSCs, enhance nerve regeneration [48]. In a direct comparison with the rabbit facial nerve, they appeared to be even more effective [52].

PLAC NGC seeded with autologous BMSCs and further reinforced with the ulnar vein was used to bridge 30 mm long-ulnar nerve defects in beagle dogs and compared to autologous nerve graft treatment. Analysis at 8 weeks confirmed the differentiation of BMSCs into SCLCs, as evidenced by glial cell marker expression. Regarding functional measurements after 12 weeks, amplitude and nerve conduction velocity were lower than the autologous group. Interestingly, measurements at 24 weeks showed a significant improvement in functional recovery, similar to the autologous group. These observations further supported enhanced axonal regeneration, myelinated–axonal number, and wet-weight muscle. Although these results are promising, important controls (role of PLAC versus vein graft for delivering the cells) are missing. Therefore, further studies are needed to evaluate their clinical potential [49]. Mongrel dogs with a 10 mm facial nerve defect received treatment involving autologous saphenous vein graft alone or in combination with autologous BMSCs or PRP. Control groups received no treatment. Functional analysis revealed BMSC treatment-dependent facial functional improvement, thus indicating its therapeutic potential. Eyelid, ear, lip, and tongue functions were higher after 8 weeks postoperatively in the BMSC group compared to all other groups. Furthermore, reduced collagen deposition and tissue adhesions were associated with a higher axonal count. However, further studies are needed for clinical translation [67].

Tracking and fate analysis of transplanted cells is a prerequisite for an understanding of host-graft interaction and the tissue regeneration process. Within this context, the ability of MRI for in vivo tracking of transplanted BMSCs was tested. For this, cells were labeled with clinically-available paramagnetic contrast agent (Gd-DTPA) and implanted into rabbit sciatic nerve after a 10 mm-long traction injury. In contrast to unlabeled cells, animals with Gd-DTPA-labelled cells exhibited signals for up to 10 days [57]. In a subsequent study, rabbits treated with BMSCs following a 10 mm-long traction injury of the sciatic nerve showed improved functional recovery in terms of toe spreading and a significantly improved index for ankle dropping [40].

The research of Trindade et al. is at the lower limit of nerve gap size. In a rabbit model, they investigated the repair of a 5 mm femoral nerve gap with a BMSC-seeded silicone tube. Although histological analysis showed no significant difference with the control group (saline), a functional assessment showed a tendency towards better recovery using BMSC-loaded NGCs [42].

Sheep represent an important animal model to facilitate extrapolation to humans. Casañas et al. used Neurolac^®^ (Polyganics, Groningen, The Netherlands), which is basically a PLAC tube, as an artificial NGC in sheep, with the addition of platelet-rich plasma (PRP) as a growth factor supplement for the treatment of a 10 mm-long defect of the tibial or radial nerve. Despite the small sample size, they observed significant differences when comparing NGCs loaded with BMSCs to PRP or NGCs alone. However, the presence of PRP had no significant effect [50].

Taken together, the reported data in the literature show that BMSC-based TENGs are promising to support peripheral nerve repair and regeneration. Nevertheless, further research is needed to establish a safe and effective protocol that can be used in the clinical setting. Furthermore, the difficulties and risks associated with BMSC isolation and culture need to be critically weighed against the benefits of other sources of MSCs.

#### 3.2.3. Other Cells and Factors Used for Nerve Tissue Engineering Applications

As bone marrow aspiration can be potentially a risky intervention, there is a clear need for an alternative and minimally-invasive access source of MSCs, such as skin-derived MSCs (SK-MSCs) from the dermis, adipose tissue-derived stem cells (ASCs), umbilical cord tissue-derived stem cells (UC-MSCs), and amniotic fluid-derived stem cells (AF-MSCs).

In 2012, Park et al. reported a study involving human SK-MSCs for the treatment of larger mammals. Using eight pigs, they showed comparable effects of SK-MSCs and BMSCs for enhancing nerve regeneration. However, as the animals were already sacrificed after two or four weeks and no functional analysis was carried out, the resulting data are insufficient to draw firm conclusions. In another study, researchers found a better regeneration of a 10 mm femoral nerve gap in SK-MSC-loaded grafts, but not more significant than in controls. Taken together, further research using SK-MSCs holds great promise in the field of nerve repair [58]. In a long-term functional assessment study, Su et al. demonstrated the recovery of motor nerve conduction of the sciatic nerve using a mini-pig nerve defect model for up to 20 months. For this, PLA NGCs loaded with human AF-MSCs were engineered. Twenty months after nerve repair, functional analysis showed the highest recovery of CMAP in the animal treated with AF-MSCs compared to the empty PLA NGC. Furthermore, fiber tractography using diffusion tensor MRI revealed an augmented axonal regeneration in the presence of AF-MSCs [62].

In contrast to other MSCs, ASCs are easily accessible in adequate quantities for cell therapy. Ghoreishian et al. studied the use of ASCs in a dog model of a 7 mm facial nerve defect. Despite bringing the proof of concept for ASC-based therapy enhancing peripheral nerve repair, the study suffers from a conduit material (GoreTex^®^) that is unable to support regeneration on its own compared to other conduit materials reported earlier. However, the addition of ASCs had a positive effect on facial nerve regeneration, but it was not significant from a histological point of view [41]. By contrast, the research of Lasso et al. showed more exciting outcome measurements. Their experimental set-up consisted of 60 rabbits with a 40 mm peroneal nerve gap bridged with human ASCs in fibrin-filled autologous veins or empty grafts as controls. In order to prevent rejection, the experimental animals and one control group were treated with cyclosporine A. Resulting data showed the highest density and thickness of axons in the experimental group [51]. In a subsequent study using autologous ASCs, a rabbit 10 mm-long sciatic nerve was repaired. For this, an acellular xenogeneic nerve graft was loaded with autologous ASCs labeled with CM-Dil and PRP. As a control, graft with or without ASCs and autologous nerve graft was applied. After 8 weeks, an analysis of transplanted cells and regenerated nerves confirmed the viability of transplanted cells with SCLC-like features and enhanced axonal growth and myelin regeneration. The addition of PRP to cell transplantation appeared to enhance the therapeutic performance comparable to autologous nerve treatment in contrast to all other groups. However, the effects of PRP alone on nerve repair are unknown and further studies are needed to evaluate the therapeutic potential of the combination of ASC and PRP on critical nerve gap injuries [64]. A further interesting contribution on this topic is from Xiao et al. In a long-segment defect, they used chitosan conduits with human UC-MSCs to attach the peroneal nerve to the tibial nerve in an end-to-side anastomosis. The tibial nerve itself then served as a scaffold for the reinnervation of the peroneal innervation field by lateral budding. Results showed that the UC-MSCs played a key role in the induction and growth of the lateral nerve bud, leading to a faster and better recovery, as indicated by significantly better electrophysiological recovery, as well as a high number of myelinated axons [44].

**Figure 4:**
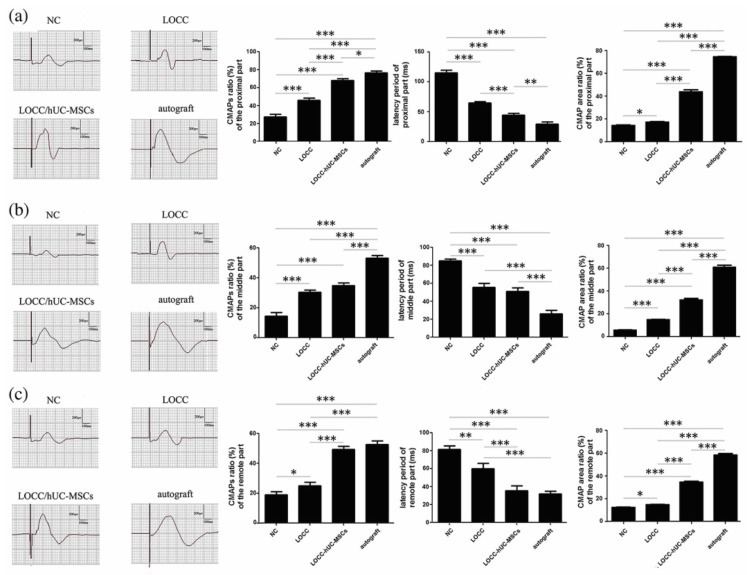
Functional collagen nerve guide consisting of longitudinally aligned fibers and human umbilical cord derived mesenchymal stem cells promote functional recovery after sciatic nerve 35 mm gap injury in dogs. Quantitative results of the electrophysiological evaluation made 9 months after surgery. Representative measurements of the (**a**) proximal, (**b**) middle, and (**c**) distal sections of the injured canine sciatic nerve. The data are shown as the mean ± standard deviation. * *p* < 0.05, ** *p* < 0.01, or *** *p* < 0.001, compared with the negative control (NC) group. cMAPs = compound muscle action potentials; hUC-MSCs = human umbilical cord mesenchymal stem cells; LOCC = longitudinally oriented collagen conduit. These figures were adapted from published work by Cui et al., 2018 [63].

UC-MSCs were also tested using a dog model of a 35 mm sciatic nerve defect. For this, longitudinally aligned collagen NGC loaded with human UC-MSCs was fabricated and implanted between the transected nerve stumps. Functional and histomorphometry analysis after 9 months of treatment showed enhanced nerve regeneration and electrophysiological recovery compared to the control group, indicating the potential of UC-MSCs for neurotrophic support and axonal regrowth (Figure 4) [63]. In a scaffold-free approach, 3D NGC was produced from autologous dermal fibroblast cells using 3D bioprinting. The resulting NGCs were used to repair a 5 mm ulnar nerve defect in beagle dogs and compared to healthy nerves. After 10 weeks, functional measurements confirmed the restoration of CMAP and motor conduction properties. However, this study lacked control experimental groups, although the outcome measurements were comparable to uncut healthy nerves [65].

SCs play an important role in nerve repair and regeneration. For assessing the therapeutic potential of human SCs, a 40 mm-long ulnar nerve injury in the monkey was used. Acellular nerve graft seeded with human SC transferred for bridging the 40 mm gap injury was compared to autologous nerve grafting. Electrodiagnostic and immunohistochemistry findings after 5 months showed a significantly enhanced functional recovery in animals treated with SCs compared to empty graft, which reached the autograft level. Cell treatment protected the animals from hypothenar muscle degeneration. Furthermore, no palmar erosion or ulcers were observed after 5 months of treatment. These results are highly promising and show the potential of SCs for critical nerve gap injuries [61].

**Figure 5:**
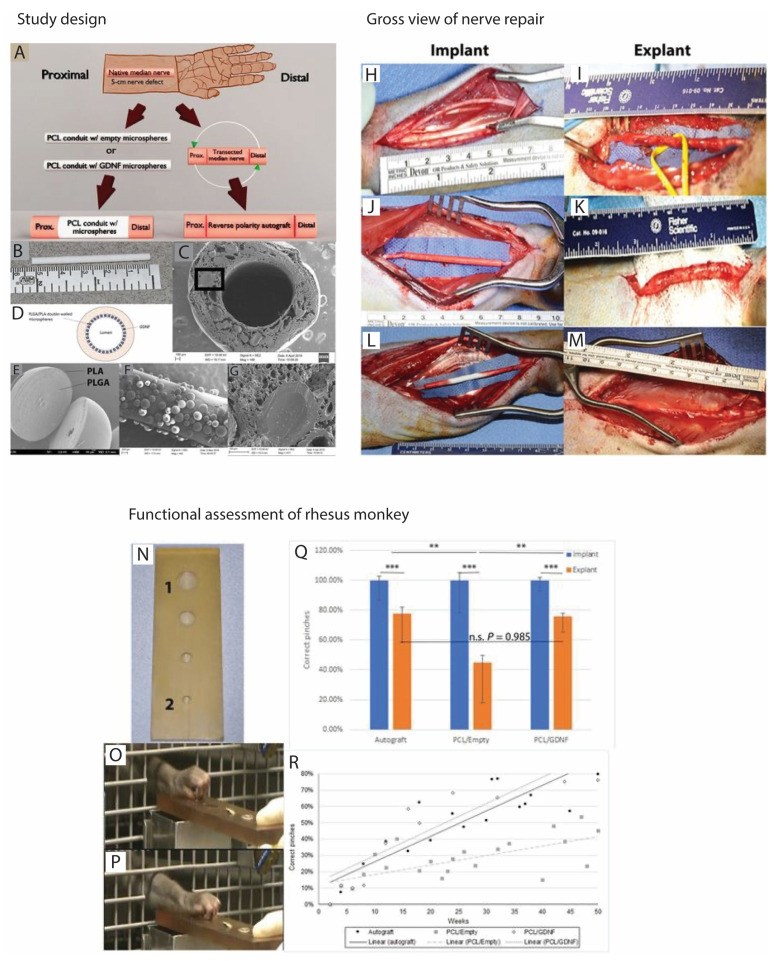
GDNF promotes long-gap nerve regeneration after 50 mm median nerve injury of rhesus monkey. Study design: (**A**) Schematic depicting experimental design. (**B**) Photograph of the 5.2 cm PCL/GDNF nerve guide. (**C**) SEM of the nerve guide cross section embedded with double-walled microspheres. Mag, magnification. (**D**) Diagram of the PCL/GDNF nerve guide cross section. (**E**) SEM of a bisected double-walled PLGA/PLA microsphere. (**F**) SEM of microsphere adhesion to the initial PCL layer during the manufacturing process. (**G**) Higher magnification of a cross section of a double-walled PLGA/PLA microsphere embedded in the PCL wall [rectangle in (**C**)]. EHT, electron high tension; WD, working distance; Photographs of (**H**) exposed native nerve. (**I**) PCL/GDNF conduit explanted after 1 year. (**J**) Implanted PCL/Empty conduit. (**K**) PCL/Empty conduit explanted after 1 year. (**L**) Implanted PCL/GDNF conduit. (**M**) Autograft explanted after 1 year. (**N**) Modified Klüver board with varying well diameters used for functional training and assessment; Well 1 has a diameter of 2.5 cm, and well 2 has a diameter of 0.5 cm. (**O**) Photograph of the correct pinching motion. (**P**) Photograph of the incorrect pinching motion. (**Q**) Normalized functional bar graph comparing the NHPs’ 50-week functional recovery to their preoperative baselines; n.s., not significant. (**R**) Linear regression plot assessing functional recovery over 50 weeks for all treatment groups. *n* = 30 measurements per time point per NHP. Means represented with +SE/−SD. Adjusted *p* values presented as: ** *p* < 0.01; *** *p* < 0.001 (select comparisons shown). These figures were adapted from published work by Fadia et al., 2020 [66].

As an alternative option to cell therapy, direct nerve lengthening and growth factors treatment was tested in a subsequent experiment. Using a direct nerve lengthening procedure, a 20 mm nerve defect in the median monkey nerve was repaired. The resulting data were compared to autologous nerve graft treatment. Functional, electrophysiological, and histological analysis after 5 months revealed a superior recovery in animals treated with direct nerve lengthening than in autologous animals. Although these results are extremely interesting, nerve lengthening is associated with increased tension and nerve stiffening, and fibrosis. Therefore, further research is needed to establish the therapeutic efficacy of this approach for critical gap injuries [59]. In a critical nerve gap repair model involving a 50 mm median nerve defect in the rhesus monkey, PCL NGC releasing GDNF promoted functional nerve regeneration comparable to autologous nerve grafting (Figure 5). Twelve months after nerve reconstruction, nerve conduction velocity was significantly improved and matched well with the autologous group. These results are in agreement with the measurements of myelinated axons, fiber density, g-ratios, and SCs in the distal part of the reconstructed nerve. These results are highly promising, and further clinical studies would enhance the application of this bioactive PCL-GDNF [66].

**Figure 6:**
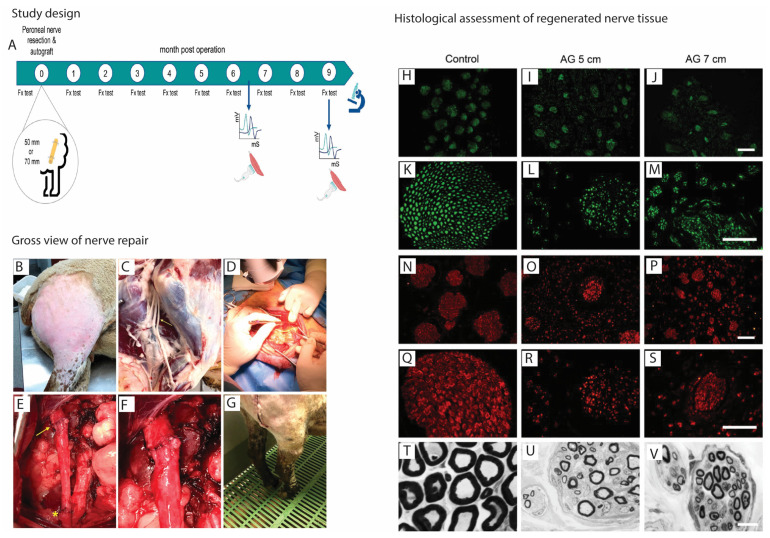
Repair and regeneration of long-peripheral nerve injuries in sheep model. (**A**) Following the surgery, functional tests (Fx test) were performed each month, electrophysiological tests and echography were made at 6.5 and 9 months, and samples were taken for histology at the end of the follow-up. (**B**) The surgical approach was performed with the animal in lateral recumbency through a lateral longitudinal skin incision. (**C**) Wide dissection showing the peroneal nerve location (arrow) after the sciatic nerve bifurcation into the tibial and peroneal nerve in a cadaveric sheep. (**D**) Resection of the common peroneal nerve under the operating microscope to create the nerve gap. (**E**) A 5 cm autograft was sutured again to the nerve stumps with epineural sutures (proximal suture marked with yellow arrow and distal suture marked with a yellow asterisk). (**F**) Detail of the 8 stitches made to join the nerve graft with the healthy nerve stump without tension. (**G**) After the surgery, some animals showed foot drop in the standing position. Representative immunohistochemical images of transverse sections of a control peroneal nerve (**H**,**K**,**N**,**Q**) and of an autograft of group AG5 (**I**,**L**,**O**,**R**) and of group AG7 (**J**,**M**,**P**,**S**). Sections were immunolabeled against NF200 for myelinated axons (**H**–**M**), and against S100 for Schwann cells (**N**–**S**). Images were taken at ×40 magnification (**H**–**J**,**N**–**P**), scale bar 200 μm, and at ×400 magnification (**K**–**M**,**Q**–**S**), scale bar 100 μm. The bottom panels show representative semithin transverse sections of the middle segment of the nerve graft stained with toluidine blue. (**T**) control nerve, (**U**) AG5, and (**V**) AG7 graft, at 10,000× magnification, scale bar 10 μm. These figures were adapted from published work by Contreras et al., 2023 [36].

For further evaluation of the therapeutic potential of functionalized nerve guides using critical nerve gap injuries, the sheep model with ≥70 mm nerve gap injury appears to be promising (Figure 6) [36].

Taken together, these studies clearly showed different MSCs as a potential source for developing TENGS to treat peripheral nerve defects. The data and observations obtained from various large animal models suggest BMSCs are the most promising therapeutic source. Likewise, ASCs also emerged as a promising cell source due to the ease of isolating an adequate number of cells using a minimally invasive process (i.e., lipo-aspiration) compared to the BMSCs’ invasive isolation procedure, coupled with the low quantity of cell numbers. Further research is needed to establish the sufficient safety profile and efficacy of ASCs in various large animal models. However, different MSCs possess largely similar therapeutic mechanisms in mediating axo-glial regeneration.

Based on the experimental evidence, MSCs promote tissue regeneration by modulating the immune-microenvironment and by secreting a wide range of growth factors [68,69]. Subsequently, transplanted MSCs differentiate into Schwann cell-like cells and contribute to the process of nerve tissue regeneration.

MSCs release a wide range of molecules (miRNAs, anti-inflammatory cytokines, and growth factors) via their secretome into the extracellular space, thereby impacting the biological response of the host tissue. MSCs secretome carrying IL-4, IL-10, and IL33 activates the STAT3 pathway, resulting in the M2 polarization of macrophages, which are known to reduce inflammation and enhance the tissue regeneration process [68]. Further, MSCs secretome releases several neurotrophic factors, which are all linked to axo-glial regeneration, such as neurotrophin-1 (NT-1), neurotrophin-3 (NT-3), neurotrophin-4 (NT4), ciliary-derived neurotrophic factor (CDNF), brain-derived neurotrophic factor (BDNF), nerve growth factor (NGF), glial cell line-derived neurotrophic factor (GDNF), fibroblast growth factor (bFGF), and ciliary neurotrophic factor (CNTF) [69]. However, recent studies show that cellular aging (i.e., donor age, increased passages, replication stress, telomere deletion, and cellular exhaustion) drives MSCs into senescence phenotype [70,71]. Resulting senescent MSCs are known to exhibit pro-inflammatory effects and impede tissue regeneration [70,71,72].

## 4. Conclusions and Future Perspectives

The current gold standard for peripheral nerve repair that cannot be repaired with end-to-end anastomosis is autografting. Considering the drawbacks, such as donor site morbidity and size and modality mismatch, there is a clear need for viable replacement options. Research in the field provides ample evidence for the excellent therapeutic efficacy of TENGs to a level matching autografts, particularly in the case of subcritical (10 mm to 30 mm) nerve gap injuries, but only a few studies have reported repair outcomes in the case of critical gap injury, i.e., up to a 60 mm-long-defect. In the field of cell therapy, BMSCs have been the subject of much research with promising results both in rodents and non-human primates. However, the bone marrow aspiration procedure is a risky intervention. Within this context, ASCs hold great promise. Although the nerve regenerative potential of ASCs has been well established in rodent models, studies evaluating their therapeutic efficacy for critical nerve injuries in large animals are limited in number, and further research is needed to pave the way for their clinical translation. Stem cell therapy could also be effective in other forms of traumatic situations, such as traction injuries and end-to-end and end-to-side anastomosis, to bridge extra-large nerve gap defects.

In this review, we have identified the following major challenges in the field of nerve trauma reconstruction, and further research addressing these issues needs to be considered in order to advance in the field. (1) The fate of transplanted cells is unclear in most studies reported in the present review, thus leading to ambiguity in drawing reliable conclusions linking the transplanted cells to outcome achievements. Further research evaluating the fate and safety, i.e., neoplasm development, of the transplanted stem cells is fundamentally required to develop more effective and advanced therapeutic strategies in order to offer viable and effective cell-based treatment options in the field of neuropathic disorders. (2) Ischemic necrosis of the transplanted cells in the severed and critical gap nerve injury defects (20–60 mm). A solution to improve the outcome in critical nerve gap defects would be the pre-vascularization of the graft using 3D-biofabrication techniques to prevent cell death. Another method to reverse ischemia-induced cell death is the slow and local release of oxygen from the scaffold materials of the NGCs. (3) Rapid immune rejection of the heterologous cells will impede the regeneration process. In order to reduce rejection, the slow and local release of immunosuppressive agents could be considered. (4) Usage of animal-origin supplements for the isolation and culture of the stem cells. Cell therapies towards clinical translation would need to consider the patient’s safety and the principles of good manufacturing practice. The risk of infection from animal-derived products is especially pertinent in addition to the undesired immune reactions. (5) Lack of off-the-shelf products, particularly in the case of autologous therapies. Clinically, one problem regarding the future use of TENGs in humans is the time period between the traumatic injury and the implantation of the therapeutic grafts. Stem cells need to be harvested, cultured, perhaps differentiated, and then seeded into the conduits before the graft can be implanted. This is why it would be potentially favorable if we could enhance the efficacy of heterologous stem cells to the level of autologous stem cells. This would allow clinical centers to establish tissue banks with pre-fabricated TENGs in different sizes that could be immediately available for transplantation. Notably, prophylactic tailor-made TENGs could be worth the cost for individuals at risk for nerve injuries, such as soldiers, carpenters, and other people working with heavy machines. This would allow for repairing nerve injuries in a faster and more effective way than the presently available clinical interventions.

In summary, TENGs involving BMSCs, ASCs, UC-MSCs, SCs, AF-MSCs, and SK-MSCs have demonstrated a high potential for significant and reproducible results for the enhancement of peripheral nerve regeneration over a range of nerve gap injuries of 10 mm to 60 mm in different animal models, such as rabbits, pigs, dogs, sheep, and monkeys. Based on a large amount of evidence from various large animal models, BMSCs appear to be the most promising therapeutic source. On the other hand, based on the ease of access and culture, ASCs hold great promise for the future. However, further research on the aforementioned challenges is needed to establish safety and efficacy and to facilitate effective clinical translation.

## Data Availability

All datasets related for this article will be provided upon request.
